# Regional Heterogeneity of Application and Effect of Telemedicine in the Primary Care Centres in Rural China

**DOI:** 10.3390/ijerph17124531

**Published:** 2020-06-24

**Authors:** Wanchun Xu, Zijing Pan, Shan Lu, Liang Zhang

**Affiliations:** 1 School of Medicine and Health Management, Huazhong University of Science and Technology, Wuhan 430030, China; wanchunxu6@gmail.com (W.X.); peterpanzijing@gmail.com (Z.P.); shanlu@hust.edu.cn (S.L.); 2 Research Centre for Rural Health Service, Key Research Institute of Humanities & Social Sciences of Hubei Provincial Department of Education, Wuhan 430030, China

**Keywords:** telemedicine, regional heterogeneity, propensity score matching

## Abstract

The increasing concerns of the geographical maldistribution of medical resources have sparked worldwide interests in exploring the potential of telemedicine in the rural health system. This study aimed to investigate the application and effect of telemedicine as well as their regional heterogeneity in the primary care centres in rural China. Based on the stratified multistage cluster sampling, a cross-sectional study was conducted among 358 township health centres (THCs) from eastern, central and western China. A self-administered questionnaire was used and the data of the Health Statistical Annual Reports in 2017 were collected to investigate the implication of telemedicine as well as the performance and other characteristics of each THCs. Propensity score matching was used to estimate the effect of telemedicine application on the bed occupancy rate and the number of annual outpatient visits of the THCs, with comparison among the regions. The overall prevalence of telemedicine application was 58.66% in 2017, and it was found to increase the bed occupancy rate of the THCs in the national range (*p* < 0.1). When divided into different regions, telemedicine was found to improve the number of annual outpatient visits in western China (*p* < 0.05) and the bed occupancy rate in eastern China (*p* < 0.1). Disparities in the degree of remoteness and the capability of THCs among the regions were also found in this study, which may be the reasons for the regional heterogeneous effects of telemedicine. These findings suggested the potential of telemedicine in improving the utilization of primary care centres in rural areas. Further studies were needed to investigate the underlying reasons for its regional heterogeneous effects.

## 1. Introduction

Due to the increasing needs of medical care, insufficient medical resources and their unbalanced allocation has become a worldwide problem [1]. The majority of health workers tend to work in urban areas, leaving a shortage of health workers in remote and rural areas, especially in the rural primary care system [2,3]. Due to the shortage of skilled doctors and limited laboratory services, some patients in remote areas suffered from misdiagnosis and delay of treatment [4]. Some other patients in rural areas tend to travel to the tertiary hospitals in urban areas, leading to the underutilization of health care facilities in rural areas and overloading in the tertiary hospitals in urban areas [5]. Increasing concerns about poor access to high-quality medical care and sustainability of primary care facilities in rural areas have sparked the worldwide interest in exploring the potential of telemedicine in the rural health system [6].

Telemedicine, as defined by WHO, refers to the delivery of health care services, where distance is a critical factor, by all health care professionals using information and communication technologies (ICTs) for the exchange of valid information for the diagnosis, treatment and prevention of disease and injuries, research and evaluation, and for the continuing education of health care providers, all in the interests of advancing the health of individuals and their communities [7]. In industrialized regions, such as the UK and the USA, the majority of telemedicine services focus on diagnosis and clinical management [8,9]. However, in the low-income countries or the regions with limited infrastructure, telemedicine applications are primarily used to link healthcare providers with specialists, referral hospitals and tertiary care centres [10].

Telemedicine was introduced into China in the 1980s [11] and has developed quickly with the rapid growth of telecommunication networks [12], which was considered as one of the strategies to cope with the geographical maldistribution of medical resources [13]. The long-lasting problem of Healthcare maldistribution [14,15] has led to the “inverted pyramid” structure of healthcare utilization in China, which means the underutilization of primary care or health facilities in rural areas, and the overcrowding of patients in tertiary hospitals in urban areas [16,17]. Apart from the shortage of health care resources, the inhabitants were much more scattered, and transportation was less developed in rural China [18,19]. Moreover, China is experiencing rapid ageing and urbanization, and the degree of ageing in rural China is higher than that in urban areas, which indicates the importance to improve the rural health system to respond to the increasing need of the elderly [20,21]. As the primary care centres in rural areas [22], a number of township health centres (THCs) have been operating in an inefficient state, including the underutilization of outpatient and inpatient services [23,24]. To improve the capacity and utilization of the primary care system in rural areas, China had highlighted the development of telemedicine in the construction of a vertically integrated health system [25]. In 2007, the telemedicine network of Gansu Province was founded, which covered all of the municipal hospitals, county hospitals as well as the THCs in the province [26]. With the development of medical informatization, the expansion of the service platform of telemedicine to the THCs was realized in more and more provinces [27,28]. The promotion of telemedicine on the township level was considered to improve the residents’ accessibility to high-quality medical service and their utilization of primary care facilities in rural areas by the policy-makers [27].

Although telemedicine was proved to improve the efficiency and quality of medical care for patients in some previous studies around the world [29,30,31,32], few studies have investigated its effect on the medical institutions or the medical system. Health institutions were considered to benefit from applying telemedicine since it could help to improve the bed occupancy or other resource utilization [33], especially for the regional health institutions in rural areas [34], but little research evidence was found on this aspect. Despite the rapid growth of telemedicine among the THCs in China, its effects on the improvement of the rural health system also remain unclear. It was reported in a survey that most administrators in the hospitals with telemedicine in China agreed that telemedicine was useful for the order of pyramid hierarchical medical system and the two-way transfer of medical treatment [26]. However, to our knowledge, the effect of telemedicine on the utilization of the primary care facilities in rural China has not been evaluated yet. Moreover, the majority of the empirical studies on telemedicine in China were based on the regional telemedicine practice, such as the Internet-based hospital in Guangdong province (eastern China) [27] and the telemedicine networks in Gansu province [26] and Guizhou province (western China) [28]. Few studies have investigated the nationwide penetration of telemedicine and its effect on the health care system. There are great disparities in the economic development, urbanization level and health care systems among different regions in China [35]. Medical resources were mainly distributed in developed eastern China, followed by the densely populated central China. However, the vast area of western China was sparsely populated, and economic development and health systems were relatively backward [36]. In such a case, it is of great significance to examine the regional heterogeneity of the effect of telemedicine, which was crucial to the development of the differential strategies of telemedicine promotion.

The gold standard for drawing inferences about the effect of a policy is the randomized controlled experiment [37]. However, it was unpractical and was not applied to the policy of telemedicine promotion in China, which has been implemented for years in some pilot areas. One of the common methods for evaluating the effectiveness of telemedicine was the propensity score matching (PSM) [38,39], which could partly correct endogeneity [40] and has been widely used in the evaluation of the policy effect on the institutions [41,42,43]. According to the literature review, not only the intrinsic factors of health care facilities, such as the size of hospitals and the provision of surgical service, but also the external factors including the medical insurance policy, the geographical accessibility to the urban hospitals and the disease spectrum in the service areas would affect the utilization and efficiency of the health facilities [44,45,46]. It is, therefore, worthwhile to collect the information above to evaluate the effect of telemedicine on the institutional level through PSM.

The present study aimed to fill the gaps mentioned above by evaluating the effect of telemedicine application on the utilization of outpatient and inpatient services of the THCs in three regions of China through the method of PSM. The number of outpatient visits and the bed occupancy rate were examined as outcome variables in the present study. Using national-scale data, the usage of the telemedicine across rural China are reported in this study, which has not been reported before as far as we know. The findings of the present study would help to provide evidence-based recommendations for the policy development of telemedicine in rural China.

## 2. Materials and Methods

### 2.1. Study Sample and Data Collection

This study was based on the data from a national survey to assess the capacity of the rural health facilities, which is a part of a research on the capacity-building of the primary care system in China. A combination of stratified multistage sampling and cluster sampling was applied in this cross-sectional survey. In the first stage, six provincial regions were selected based on the socio-economic level and geographic distribution (eastern China: Shandong, Guangdong; central China: Henan, Hubei; western China: Guizhou and Chongqing). In the second stage, one developed city and one less-developed city (based on the gross regional product (GRP) per capita) were randomly selected in each provincial region. In the third stage, given the number of counties (representing rural areas) was twice that of districts (representing urban areas) in China, two counties (a developed one and a less-developed one according to the GRP) and one district were randomly selected in each city [47]. In particular, two districts from Shenzhen City and four counties from Shaoguan City in Guangdong Province were selected since there is no county in Shenzhen City; two districts and four counties were sampled in Chongqing provincial region, which was a provincial-level city directly under the Central Government of China. In total, 24 counties and 12 districts were selected. Lastly, all the primary care facilities in the sampled counties/districts were investigated in the research project and the THCs in 24 counties were analysed in the present study.

The data were collected through a survey in collaboration with the local health bureau in each selected county from October to November in 2018. The local health bureaus helped to initiate the contact between the study team and the local THCs online. The administrators who were accessible to the related information in the sampled THCs were asked to fulfil the self-administered questionnaires, which included the questions on the implementation of telemedicine and other information of the THCs as well as their service areas. Besides, the data of the performance of the sampled THCs were downloaded from the National Health Statistics Reporting System, in which the health care facilities are required to submit a yearly report of its resources and volume of services at the end of each year [47].

In total, 405 THCs in the selected counties participated in the survey. The health Statistical Annual reports in one of the counties in Central China were missing. Cases with missing values on some variables in the present study were excluded from the analyses, and the final sample size was 358.

This study was approved by the Ethics Committee of Tongji Medical College, Huazhong University of Science and Technology (IORG No: IORG0003571).

### 2.2. Study Design and Statistical Variables

We used a matched pair design with propensity score matching (PSM) in the present study to analyse the correlation between the application of telemedicine and the utilization of THCs. Using observed characteristics, PSM constructs a statistical comparison group that is based on a model of the probability of participating in the treatment. The average treatment effect of the program is then calculated as the mean difference in outcomes across these two groups [48]. The sampled THCs were asked if they had provided telemedicine services in the care for patients in 2017 (yes/no). According to the government document on the development of telemedicine by the national health commission of China [49], the telemedicine service was defined as the medical practice in which a medical institution invites other medical institutions to provide technical support for the diagnosis and treatment for the patients through the information and communication technologies after signing the telemedicine cooperation agreement. All the respondents were informed of this official definition. The THCs that answered “yes” to the question above were defined as the treatment group, and those with the answer of “no” were considered as the control group.

There were two outcome indicators in the present study. One is the number of outpatient visits of each THCs in 2017, which was used to show the annualized utilization of the outpatient service of a hospital or the other health institution [47,50]. The other one is the bed occupancy rate in 2017, which was calculated as the number of beds effectively occupied (bed-days) divided by the number of beds days available in 2017, and this indicator is often used to show the actual utilization of an inpatient health facility for a given period [51].

Variables of the characteristics of the THCs and their service areas were included in the propensity score models to reduce the bias. According to the literature review, the covariates in the propensity score model for the number of annual outpatient visits were selected in the context of the present study as followed: (1) population size in the service area of each THC; (2) the proportion of the elders (≥65-year-old) in the regional service population in 2017(according to data of public health services in the Health Statistic Annual Report); (3) the number of the staff in each THC; (4) travel time to the county seat by bus (≤1 h/>1 h); (5) global budget for county medical alliance (yes/no) [52,53,54,55]. As for the model for bed occupancy rate, covariates included the variables above with another variable added: (6) the provision of surgical service (yes/no) [56,57].

### 2.3. Statistical Analysis

Description analysis of the study variables mentioned above was used to demonstrate the telemedicine application and characteristics of the overall sampled THCs and those in each region of China respectively (eastern, central and western China). The telemedicine adoption in the THCs in each province was also calculated.

PSM was used to evaluate the effect of telemedicine on the number of annual outpatient visits and bed occupancy rate of the THCs across China and in each region. Firstly, the conditional probability of applying telemedicine of each sampled township hospital was calculated using a logit model to derive the propensity scores. The propensity score can be estimated by the parameter estimation method (such as probit or logit model) and nonparameter estimation method (such as discriminant analysis). For the binary treatment case, where we estimate the probability of participation vs nonparticipation, logit and probit models usually yield similar results, so the choice of the method is not too critical. Secondly, the THCs in the treatment and control groups are matched based on the propensity score. We employed three different matching algorithms to confirm the robustness of estimates, which were the nearest-neighbour matching (NNM), radius-based matching (RBM) and kernel-based matching (KBM) separately. NNM method is one of the most frequently used matching techniques, where each treatment unit is matched to the comparison unit with the closest propensity score. To reduce the risk of bad matches in the method of NNM (if the closet neighbour is far away), a tolerance level is imposed on the maximum propensity score distance (calliper) in the method of RBM. The recommended calliper in RBM was no more than 25% of the standard deviation of the propensity score of the sampled subjects [48,58]. According to the calculation, the standard deviation of the propensity score in the models for the number of outpatient visits was 0.126 (0.124, 0.131 and 0.123 in the group of eastern, central and western China respectively). In the models for bed occupancy rate, the standard deviation of the propensity score was 0.138 (0.134, 0.137 and 0.135 in the subgroups mentioned above respectively). Therefore, the calliper was fixed at 0.02 in all the models of RBM methods. In the method of KBM, a weighted average of all nonparticipants was used to construct the counterfactual match for each participant to avoid the small size of the subset of nonparticipants that ultimately satisfy the criteria to fall within the common support [48]. These three matching algorithms were frequently used in the previous studies and showed good reliability [58]. Finally, the average treatment effect on the treated (ATT) of telemedicine on outcome variables, which were the number of annual outpatient visits and bed occupancy rate in this article, was examined based on the matched samples by *t*-test. The standard error reported by matching result did not consider the fact that the propensity score was estimated (that is, assuming the propensity score is the true value, and then deducing the standard error (S.E.)). Therefore, the bootstrap method was used to obtain a more accurate S.E. and 95% CI (confidence interval) for the ATT in the present study, which was often used in the previous studies [59]. In addition, an overall balancing test was performed to verify the reduced sampling bias achieved through matching, which would show the matching quality of each matching algorithm. Lower pseudo R^2^, insignificant *p*-value of the likelihood ratio, and the value of mean absolute standard bias (MASB) that was no larger than 20% after matching would qualify the matching process [60,61].

The statistical analysis was conducted in Stata version 12 (StataCorp LP., College Station, TX, USA).

## 3. Results

### 3.1. Application of Telemedicine among the Investigated Township Health Centres

Three-hundred and fifty-eight township hospitals were analysed in the present study, and 174 of them were from western China. Among the overall investigated THCs, 58.66% of the THCs reported providing telemedicine service in 2017. However, this proportion varied among the regions and provinces in 2017. On the regional level, 68.39% of the THCs had adopted telemedicine in western China, which was higher than those in the central (53.26%) and the eastern (45.65%) regions. The proportion of telemedicine adopters was higher in the less developed province in each region, including Shandong in eastern China (68.97%), Henan in central China (63.64%), and Guizhou in western China (91.07%). More detailed information was presented in Table 1.

### 3.2. Characteristics of the Township Health Centres in Different Regions and between Groups

Our data showed that the THCs among the regions were different in a lot of aspects (Table 2). The average population size of the service areas was the largest in the central regions, with the eastern regions followed, and the smallest in the western regions. However, the proportion of the elders in the population was higher in the western regions. As for the transportation conditions, nearly half of the THCs in the western region reported that the travel time to the county seat was more than an hour (by bus), but this proportion was only about 10% in the eastern and central region. In terms of the size and capacity of the THCs, the average number of staffs in the THCs was the largest in the eastern region, while more THCs in central regions were able to provide surgical service (67.39%) and have the higher bed occupancy rate (73.80%) and larger number of annual outpatient visits (62,170 visits in 2017). The bed occupancy rate was the lowest in the eastern region (47.55%), and the number of annual outpatient visits was the smallest in the western region (18,310 visits in 2017).

The comparison of the characteristics between the telemedicine adopters and non-adopters (THCs) were presented in Table 3. The disparities between the adopters and non-adopters further justified the including of these covariates in the PSM model. The population size of the service areas of THCs was larger in the telemedicine adopters in the overall sample (*p* = 0.048), and so as in the eastern region (*p* < 0.001). The proportions of the elders in the service areas of THCs were lower in the treatment group in the overall sample (*p* = 0.073) and those in the central (*p* = 0.044) and western areas (*p* < 0.001), but were higher in the treatment group in the eastern region (*p* < 0.001). The numbers of staffs in the THCs were larger among the telemedicine adopters in the eastern region (*p* = 0.007) and but smaller among those in the central region (*p* = 0.006). Minor differences existed but was not significant between groups in terms of the travel time to the county seat by bus and the implementation of the global budget for county medical alliances, which would still be included in the PSM models because they could influence the utilization of the service in the THCs in theory according to the literature review mentioned above.

### 3.3. Effect of Telemedicine on Number of Annual Outpatient Visits and Bed Occupancy Rate

Table 4 demonstrated the result of PSM and the overall balancing test. An overall balance test of the distribution of these variables between the telemedicine adopters and non-adopters after PSM were conducted to evaluate the bias control of each matching algorithm. In the PSM models for the ATT of telemedicine application as estimated by NNM, RBM and KBM algorithms, all the matching algorithm largely reduced the mean and median of the absolute standardized bias of the covariates (MABS < 20%), especially the algorithm of kernel matching. The pseudo R^2^ was reduced, and the *p*-value of the likelihood ratio became insignificant after matching, which indicated that the balancing property was satisfying.

In the overall sample from six provinces, telemedicine application was found to increase the bed occupancy rate of the THCs by about 6% with a lower significance (*p* = 0.082 in RBM method and *p* = 0.081 in KBM method). However, this result was not significant in NNM method, indicating that the result was not robust and should be interpreted with caution. As for the number of annual outpatient visits of THCs, no significant difference was found between the telemedicine adopters and non-adopters in the overall sample, which was consistent across all the match algorithms.

Regional heterogeneity was found in the effect of telemedicine on the utilization of THCs. In the eastern region, telemedicine application was found to increase the bed occupancy rate of the THCs by 15.20% with a lower significance (*p* = 0.093 in KBM method), but no significant effect was found on the number of annual outpatient visits. However, in western China, it was found that telemedicine could increase the number of annual outpatient visits by about one-third, which was robust among all the match algorithms (*p* < 0.05 in NNM, RBM and KBM method), but no significant difference was found in terms of bed occupancy rate. In central China, no significant difference was found of both number of annual outpatient visits and bed occupancy rate between the telemedicine adopters and non-adopters. 

## 4. Discussion

### 4.1. Application of Telemedicine among the THCs and Regional Disparities

Telemedicine has been developing rapidly with regional disparities in the progress across rural China. It was found in our study that the proportion of the THCs adopting telemedicine was 58.66% on the nationwide range in 2017. It was reported in a study based on the national-scale sample that 12.77% of the THCs had applied the telemedicine informatization system in 2013 [62]. The past few years had witnessed the rapid growth of telemedicine service in remote rural China. However, according to the results of our study, the coverage of telemedicine in THCs was much higher in the western region, with the central region following and the lowest in the eastern region. The prevalence of telemedicine adoption also tended to be higher in the less-developed province in each region. These results indicated the regional disparities in the progress of telemedicine promotion across China. Actually, the Chinese government had promised to provide more support for the poverty-stricken areas in the central and western regions in the development of Internet-based medical system, including the ICT infrastructures for telemedicine [63]. For example, as the pilot of telemedicine promotion in poverty-stricken regions, Guizhou province (in western China) had finished the construction of telemedicine service network which covered all the primary care centres at the end of 2017 [64]. The equalization of the coverage of telemedicine among the regions had already been accelerated in recent years. According to the National Health Commission, telemedicine would cover all the county medical alliances in late 2018 and will cover all the primary care facilities in the medical alliances in late 2020 [65].

### 4.2. Effects of Telemedicine on the Inpatient and Outpatient Service of THCs

The improvement of the bed utilization of THCs was one of the aims of telemedicine promotion policy in rural China [13,63]. The average bed occupancy rate of the investigated THCs in the present study was 63.42%, which was similar to that in the national yearbook of health statistics (61.3% in 2017 on the national range). This figure was significantly lower than that of the general hospitals (86.0% in 2017 in yearbook), especially the tertiary hospitals in urban areas (98.6%) [28], indicating the gap of the resource utilization between the general hospitals and the rural health facilities. We found that telemedicine would increase the bed occupancy rate of the THCs by about 6% in the overall sample in the present study. It was found in the previous studies that telemedicine could improve patients’ utilization of local facilities and reduce the bed usage rates or congestion in the emergency departments in the USA [66,67], of which the overcrowding situation was similar to that in the general hospitals in China mentioned above. According to the National Health Committee, the bed occupancy rate of the community hospitals (community health centres and THCs meeting specific standards) should be no less than 75% [68]. Compared to the average level mentioned above, the increase of 6% in the bed occupancy rate of the THCs by telemedicine was a substantial contribution to this goal. Nevertheless, the lower significance and inconsistence of the result among the matching algorithms suggested that further study were needed to verify the effect of telemedicine on bed utilization in rural health facilities.

No significant difference was found in the number of outpatient visits of the THCs between the telemedicine adopters and nonadopters in the overall sample. Although the telemedicine has been widely used between the primary care system and other health care providers for specialist opinions or referral purpose around the world, few studies have investigated the effect of telemedicine on the utilization of the outpatient services of primary care facilities [69]. Nevertheless, telemedicine has been proved to be an effective way to safely conduct outpatient visits with high patient satisfaction, especially for the departments of paediatrics [70] and rehabilitation [71,72]. It was also reported in China that the annual average prescription price through telemedicine was much lower than that in the tertiary and secondary hospitals [27]. The insignificant result in the overall sample could be partly due to the mixed context of the regions in the national range [73], which might shape the health care need and health-seeking behaviours of the residents [74,75], as well as the influence the effect of telemedicine on the utilization of outpatient service in THCs.

### 4.3. Regional Heterogeneity of the Effect of Telemedicine on the THCs

One of the key points of the present study is to investigate the regional heterogeneity of the effect of telemedicine on the THCs. As for the outpatient service, we found that telemedicine application could increase the utilization of the outpatient service of THCs in the western region, but no significant effect was found in the eastern and central regions. The effect of telemedicine on the utilization of outpatient departments of THCs may be influenced by the degree of remoteness of the local areas, which would determinate residents’ accessibility to the alternative health institutions, such as the local county hospitals or the general hospitals in the urban areas. It was reported in China that the number of visits that the patients with chronic diseases would pay to the outpatient department of the general hospitals would decrease with the increase of their travel time to the hospitals [76]. In the present study, we found that it took more than one hour to travel to the county seat by bus for half of the THCs in the vast and mountainous western China. The telemedicine in THCs in such areas may be more attractive to the residents who need the outpatient service for minor discomforts or the follow-up visits for chronic diseases [69]. On the contrary, the transportation in the eastern and central regions was much more developed, and the travel time to the county seat for about 90% of the THCs was no more than one hour, according to the results of our study. It can be inferred that the better accessibility to county hospitals or other health institutions in the eastern and central regions may impair the effect of telemedicine application on the utilization of the outpatient services in the THCs.

As for the inpatient service, telemedicine was found to improve the bed utilization in the eastern region by 15.20%, which was much higher than that in the overall sample. No significant effect was found in the western and central regions. Since the inpatient service is more demanding in the medical resources than the outpatient services, we inferred that the improvement of the utilization of inpatient services of THCs by telemedicine application might be restricted by the capacity of the THCs. According to the results of our study, the capacity of THCs was weaker in the western region in terms of the number of medical staff (nearly half of those in other regions) and the ability to provide surgery services (only 35.63% of them were able to provide surgery service). However, the bed occupancy rate in the western region was higher than the average level of the three regions. It can be inferred that the telemedicine application can hardly increase the volume of the inpatient service in these THCs with limited human resource and no qualified working conditions for surgery services. However, the THCs in the eastern region had many more workers but a low bed occupancy rate at 47.55%. It was reported that the annual average bed occupancy rate of all kinds of health institutions in eastern China was similar to those in central and western China [77], suggesting that relative overload of the inpatient departments in the general hospitals in the eastern region. The collaboration between the THCs and general hospitals through the telemedicine may help to promote the rational triage of the patients, which would encourage the utilization of the inpatient services in the THCs of higher capacity and with more idle resources in the eastern regions [78]. As for the THCs in the central region, the numbers of staff were similar to those in the eastern region, but the number of outpatient visits and the bed occupancy rate was much higher than the latter according to the results of this study. The relatively full loading status of the THCs in the densely populated central region would restrict the effect of telemedicine on the volume of inpatient services in the THCs.

### 4.4. Implications

The results of the present study have several significant implications from a policy perspective. Telemedicine was considered as a path to a vertically integrated health system by policy-makers. Despite the rapid growth of telemedicine in the primary care system in rural China, little is known about its effects on the primary care centres and regional heterogeneity. Based on the national-scale representative data, the present study has verified that telemedicine application could help to increase the bed utilization of the THCs in rural China, suggesting the potential of telemedicine in the development of the hierarchical health system [26,33]. Besides, it was found in the present study that regional heterogeneity existed in the effect of telemedicine on the THCs, which could help us to identify the potential influencing factors or major constraints of the effect of telemedicine in the specific context. Differential strategies for the further development of telemedicine in rural China are needed according to the result of our studies, which may relate to the other supporting policies for THCs. For example, the improvement of medical resources would help to promote telemedicine for inpatient services in the THCs in western and central regions [63]. The incentives for the utilization of the telemedicine outpatient service in THCs, such as reimbursement policies in medical insurance [79], are needed for residents in the eastern and central regions, who have better accessibility to the alternative medical institutions of higher levels.

### 4.5. Limitations

Our study has several limitations which mean the conclusions in the present studies should be interpreted with caution. The matching method used in the present study is based entirely on the observed characteristics, and hidden bias may exist in the conclusion, such as the differences of quality of telemedicine services among the THCs and their pricing among the areas [80]. Therefore, the analysis in this study could only provide a causal inference on the effect of telemedicine on the THCs to a limited extent. Besides, although the regional heterogeneity was found to exist in the effect of telemedicine on THCs, the relationship between the potential contextual factors and the effect of telemedicine was not examined in the present study. Further studies are needed to investigate the impact of some contextual factors on the utilization of telemedicine inpatient and outpatient services in the THCs, which may help to reveal the underlying reasons for the regional heterogeneous effect of telemedicine on THCs.

## 5. Conclusions

Based on the geographically representative survey data, we have found that 58.66% of the township health centres in rural China applied telemedicine in 2017, and this proportion was much higher in western China, with the central region following and the lowest in the eastern region. In each region, the prevalence of telemedicine adoption also tended to be higher in the less developed province. Using PSM, telemedicine application was found to increase the bed utilization of the investigated THCs but have no impact on the outpatient service utilization of the THCs on the national range. Regional heterogeneity was found to exist in the effect of telemedicine on the THCs. According to the results, telemedicine could increase the utilization of outpatient services in western China and inpatient services in eastern China, and no significant impact was found for the central region. The regional heterogeneity of the effect of telemedicine may be due to the disparities in residents’ accessibility to alternative medical institutions and the capability of THCs among the regions. Further studies are needed to investigate the underlying reasons for the regional heterogeneous effects of telemedicine. Differential strategies for the promotion of telemedicine and other supporting policies are needed for THCs in different regional contexts.

## Figures and Tables

**Table 1:** Application of telemedicine among the investigated township health centres (THCs).

	Province	Number of THCs	THC with Telemedicine	Percent (%)
Overall		358	210	58.66
Eastern region		92	42	45.65
	Guangdong	34	2	5.88
	Shandong	58	40	68.97
Central region		92	49	53.26
	Hubei	37	14	37.84
	Henan	55	35	63.64
Western region		174	119	68.39
	Chongqing	118	68	57.63
	Guizhou	56	51	91.07

**Table 2:** Characteristics of the investigated township health centres in different regions (frequency/mean(percent/SD)).

Variables	Overall	Eastern	Central	Western	*p*
Travel time to the county seat					
≤1 h	255 (71.23%)	83 (90.22%)	82 (89.13%)	90 (51.72%)	<0.001
>1 h	103 (28.77%)	9 (9.78%)	10 (10.87%)	84 (48.28%)	
Global budget for CMA ^1^					
yes	73 (20.39%)	10 (10.87%)	17 (18.48%)	46 (26.44%)	0.010
no	285 (79.61%)	82 (89.13%)	75 (81.52%)	128 (73.56%)	
Surgical service provision					
yes	162 (45.25%)	38 (41.40%)	62 (67.39%)	62 (35.63%)	<0.001
no	196 (54.75%)	54 (58.70%)	30 (32.61%)	112 (64.37%)	
Population size (in thousand) ^2^	29.13 (29.53)	35.89 (21.55)	40.10 (35.16)	19.75 (26.99)	<0.001
Proportion of the elders (%)	11.72 (3.41)	10.99 (1.88)	10.59 (3.72)	12.71 (3.58)	<0.001
Number of the staff	43.70 (32.85)	56.99 (34.95)	55.79 (38.61)	30.28 (21.16)	<0.001
Bed occupancy rate (%)	63.42 (26.58)	47.55 (24.28)	73.80 (22.47)	66.32 (26.14)	<0.001
Outpatient visits (in thousand) ^3^	31.54 (37.86)	25.94 (23.82)	62.17 (57.73)	18.31 (14.64)	<0.001

Note: ^1^ CMA: county medical alliance. ^2^ Population size (thousand): the number of the residents in the service area of the township health centre. ^3^ Outpatient visits (thousand): the number of outpatient visits of the THCs in 2017.

**Table 3:** Characteristics of the investigated township health centres between groups in different regions (frequency/mean ± percent/SD).

Variables	Classification	Adopters ^1^	Nonadopters ^2^	*p*
Travel time ^3^ (>1 h)	Overall	64 (30.48%)	39 (26.35%)	0.396
	East	2 (4.76%)	7 (14.00%)	0.137
	Central	7 (14.29%)	3 (6.98%)	0.261
	West	55 (46.22%)	29 (52.73%)	0.424
Global budget for CMA ^4^ (yes)	Overall	47 (22.38)	26 (17.57%)	0.266
	East	6 (14.29%)	4 (8.00%)	0.335
	Central	8 (16.33%)	9 (20.93%)	0.570
	West	33 (27.73%)	13 (23.64%)	0.569
Surgical service provision (yes)	Overall	103 (49.05%)	59 (39.86%)	0.086
	East	27 (64.29%)	11 (22.00%)	<0.001
	Central	32 (65.31%)	30 (69.77%)	0.649
	West	44 (36.97%)	18 (32.73%)	0.586
Population size (in thousand) ^5^	Overall	31.71 (2.48)	25.46 (1.31)	0.048
	East	49.25 (3.23)	24.68 (2.07)	<0.001
	Central	41.22 (6.67)	38.83 (2.06)	0.747
	West	21.61 (2.91)	15.72 (1.43)	0.182
Proportion of the elders (%)	Overall	11.72 (0.18)	12.11(0.28)	0.073
	East	11.71 (0.34)	10.38 (0.18)	<0.001
	Central	9.86 (0.53)	11.42 (0.55)	0.044
	West	12.02 (0.31)	14.22 (0.47)	<0.001
Number of the staff	Overall	41.78 (2.20)	46.42 (2.80)	0.189
	East	67.57 (5.17)	48.10 (4.80)	0.007
	Central	45.53 (5.45)	67.49 (5.51)	0.006
	West	31.13 (1.98)	28.42 (2.73)	0.433
Bed occupancy rate (%)	Overall	66.10 (1.75)	59.61 (2.29)	0.022
	East	57.69 (3.67)	39.04 (3.03)	<0.001
	Central	72.00 (3.40)	75.86 (3.19)	0.414
	West	66.65 (2.37)	65.60 (3.63)	0.807
Outpatient visits (in thousand) ^6^	Overall	29.83 (2.15)	33.97 (3.76)	0.310
	East	26.86 (2.27)	25.17 (4.17)	0.736
	Central	53.97 (7.33)	71.52 (9.70)	0.147
	West	20.94 (1.43)	12.60 (1.41)	<0.001

Note: ^1^ Adopters: telemedicine adopters. ^2^ Nonadopters: telemedicine nonadopters. ^3^ Travel time: travel time to the county seat by bus. ^4^ CMA: county medical alliance. ^5^ Population size (thousand): the number of the residents in the service area of the township health centre. ^6^ Outpatient visits (thousand): the number of outpatient visits of the THCs in 2017.

**Table 4:** Results of propensity score matching.

Outcome Indicators	Matching Algorithm	Mean of Indicators		Results of ATT					Quality of Matching				
		Treatment	Control	ATT	*t*	S.E. ^1^	*p* > z ^1^	95% CI ^1^	Mean Bias	Media Bias	Pseudo R^2^	LRχ ^2^	*p* > χ ^2^
Overall													
NOV ^2^	Raw ^3^	29.83	33.97	−4.13	−1.02				15.4	14.1	0.051	24.90	<0.001
	NNM	29.49	30.99	−1.50	−0.26	5.56	0.787	(−12.40, 9.39)	8.7	8.6	0.007	4.30	0.545
	RBM	29.24	31.06	−1.83	−0.38	3.62	0.614	(−8.93, 5.27)	3.9	3.6	0.002	1.40	0.924
	KBM	29.49	31.81	−2.32	−0.49	3.38	0.493	(−8.95, 4.31)	3.1	3.7	0.002	0.96	0.966
BOR ^4^(%)	Raw	66.10	59.61	6.50	2.29				15.9	16.3	0.061	29.42	<0.001
	NNM	66.20	60.72	5.48	1.31	0.05	0.234	(−0.11, 12.77)	7.7	5.8	0.013	7.12	0.310
	RBM	66.20	60.34	5.85	1.72	0.03	**0.082**	(−1.06, 12.77)	3.7	3.0	0.002	1.10	0.981
	KBM	66.20	60.46	5.74	1.76	0.03	**0.081**	(−0.2, 11.71)	3.1	3.2	0.001	0.79	0.992
Eastern China													
NOV	Raw	26.86	25.17	1.70	0.34				63.8	57.7	0.374	47.42	<0.001
	NNM	23.83	32.52	−8.69	−0.73	12.01	0.469	(−32.22, 14.84)	14.7	9.2	0.060	4.80	0.570
	RBM	24.49	37.56	−13.07	−1.27	12.44	0.294	(−37.46,11.32)	13.8	12.5	0.018	0.970	0.965
	KBM	23.83	33.13	−9.30	−1.13	8.89	0.296	(−26.72, 8.13)	7.6	6.3	0.009	0.740	0.981
BOR (%)	Raw	57.69	39.04	18.65	3.95				68.7	65.7	0.405	51.37	<0.001
	NNM	56.81	42.50	14.31	1.73	0.09	0.117	(−4.05, 32.68)	17.2	20.2	0.060	5.36	0.498
	RBM	53.14	34.84	18.29	2.46	0.12	0.119	(−4.82, 41.41)	22.4	24.4	0.086	5.24	0.513
	KBM	56.77	41.57	15.20	2.02	0.09	**0.093**	(−3.69, 34.09)	14.3	13.7	0.062	5.35	0.500
Central China													
NOV	Raw	53.97	71.52	−17.55	−1.46				28.8	23.6	0.167	21.18	0.001
	NNM	53.11	52.97	0.14	0.01	13.55	0.992	(−26.41, 26.69)	5.8	6.0	0.008	1.02	0.961
	RBM	55.53	51.66	3.87	0.33	16.87	0.819	(−29.21, 36.95)	8.1	6.1	0.030	3.41	0.637
	KBM	53.11	54.98	−1.87	−0.18	12.25	0.879	(−25.87, 22.13)	5.7	6.2	0.010	1.25	0.940
BOR (%)	Raw	72.00	75.86	−3.86	−0.82				25.6	17.7	0.172	21.83	0.001
	NNM	72.66	76.79	−4.13	−0.69	0.08	0.626	(−21.49, 13.23)	14.5	13.0	0.041	5.31	0.504
	RBM	73.84	74.82	−0.97	−0.16	0.09	0.914	(−19.18, 17.22)	10.1	9.8	0.029	3.43	0.754
	KBM	72.66	76.47	−3.81	−0.66	0.08	0.620	(−19.75, 12.14)	8.5	8.1	0.019	2.45	0.874
Western China													
NOV	Raw	20.94	12.60	8.34	3.61				24.8	13.0	0.087	19.00	0.002
	NNM	20.70	14.80	5.90	2.04	2.84	**0.038**	(0.33, 11.5)	12.5	12.9	0.047	14.83	0.011
	RBM	20.79	15.28	5.51	2.07	2.50	**0.027**	(0.61, 10.41)	9.0	9.7	0.028	8.68	0.123
	KBM	20.59	15.06	5.53	2.34	2.15	**0.010**	(1.31, 9.75)	6.4	8.5	0.018	5.57	0.350
BOR (%)	Raw	66.65	65.60	1.05	0.25				22.1	13.0	0.088	19.20	0.004
	NNM	66.92	68.65	−1.73	−0.29	0.07	0.769	(−16.03, 11.73)	10.3	8.4	0.035	10.62	0.101
	RBM	66.84	69.08	−2.23	−0.38	0.07	0.746	(−14.97, 10.50)	6.3	6.3	0.017	4.74	0.577
	KBM	66.96	66.03	0.93	0.18	0.06	0.873	(−10.81, 12.68)	5.6	6.5	0.015	4.45	0.616

Note: ^1^ The standard error, *p*-value and 95% CI of the average treatment effect on the treated (ATT) were obtained through the bootstrap method. ^2^ NOV: number of annual outpatient services (in thousands). ^3^ Raw: raw sample. ^4^ BOR: bed occupancy rate.
